# Regulatory mechanism of vulnerability disclosure behavior considering security crowd-testing: An evolutionary game analysis

**DOI:** 10.1371/journal.pone.0304467

**Published:** 2024-06-21

**Authors:** Liurong Zhao, Xiaoxi Yu, Xinyu Zhou

**Affiliations:** School of Economics and Management, Nanjing Tech University, Nanjing, Jiangsu, China; Sichuan Agricultural University, CHINA

## Abstract

The security crowd-testing regulatory mechanism is a vital means to promote collaborative vulnerability disclosure. However, existing regulatory mechanisms have not considered multi-agent responsibility boundaries and stakeholders’ conflicts of interest, leading to their dysfunction. Distinguishing from previous research on the motivations and constraints of ethical hacks’ vulnerability disclosure behaviors from a legal perspective, this paper constructs an evolutionary game model of SRCs, security researchers, and the government from a managerial perspective to propose regulatory mechanisms promoting tripartite collaborative vulnerability disclosure. The results show that the higher the initial willingness of the three parties to choose the collaborative strategy, the faster the system evolves into a stable state. Regarding the government’s incentive mechanism, establishing reward and punishment mechanisms based on effective thresholds is essential. However, it is worth noting that the government has an incentive to adopt such mechanisms only if it receives sufficient regulatory benefits. To further facilitate collaborative disclosure, Security Response Centers (SRC) should establish incentive mechanisms including punishment and trust mechanisms. Additionally, publicity and training mechanisms for security researchers should be introduced to reduce their revenue from illegal participation, which promotes the healthy development of security crowd-testing. These findings contribute to improving SRCs’ service quality, guiding security researchers’ legal participation, enhancing the government’s regulatory effectiveness, and ultimately establishing a multi-party collaborative vulnerability disclosure system.

## 1 Introduction

With the rapid development of information technologies such as 5G, AI, and blockchain, the emergence of new vulnerabilities is accelerating. According to the report “Vulnerability and Threat Trends in 2023” from Skybox Security, the National Vulnerability Database (NVD) added 25,096 vulnerabilities in 2022, which increased by 25 percent year-on-year. With the ever-growing cybersecurity vulnerabilities, governments around the world encourage the discoverers to engage in discovering, reporting, verifying, patching, and releasing vulnerabilities. These processes of vulnerability disclosure aim to help other organizations in rapidly identifying and addressing vulnerabilities in real-time. However, with the expansion of hacker attacks and diversification of attack methods, more and more enterprises are choosing non-disclosure or irresponsible disclosure due to a lack of capability. In response to these challenges, a burgeoning security crowd-testing service has arisen with the aim of bolstering organizations’ vulnerability disclosure capabilities.

Security crowd-testing refers to the vulnerability testing service presented in “crowdsourcing” in the field of cybersecurity. In this process, enterprises establish a Security Response Center (SRC) first, followed by the issuance of security testing tasks with bounties according to the severity and complexity of vulnerabilities. Subsequently, security researchers, such as professionals inside the enterprises and white-hat hackers from the community, are employed to test the systems’ cybersecurity to discover exploitable vulnerabilities in software or hardware, ultimately receiving the bounties from SRC [1]. This open and innovative model breaks the constraints of traditional cybersecurity management that not only rely on internal but also external security researchers, which shortens vulnerability disclosure time and significantly increases the probability of discovering vulnerabilities [2].

**Fig 1:**
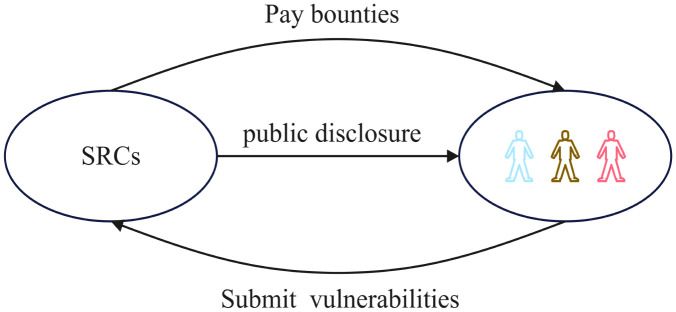
Vulnerability disclosure process under security crowd-testing.

The groundbreaking event in the field of security crowd-testing occurred in 2016 when the U.S. Department of Defense (DoD), in collaboration with HackerOne, initiated the “Hack the Pentagon” campaign, allowing external security researchers to test security vulnerabilities in certain publicly accessible websites of DoD [1]. Subsequently, large enterprises such as Microsoft, Facebook, Google, Tencent, etc. also commenced their efforts to address cybersecurity vulnerabilities by SRCs. Generally, these enterprises have well-established business models, strong technical expertise, and efficient platform operation capabilities. Their business operations are extensive with substantial volumes of sensitive data. If their cybersecurity vulnerabilities are exploited, it could result in incalculable losses. Hence, these SRCs are not only organizers of security crowd-testing but also consumers of these services. Fig 1 illustrates the vulnerability disclosure process in security crowd-testing.

However, this process involves frequent interactions among multiple participants and various resources, leading to a series of real-world issues. Firstly, the goals of participants in vulnerability disclosure are different, and a consensus on collaborative vulnerability disclosure has not yet been reached. Secondly, since all participants seek to maximize their interests, this may lead to conflicts of interests that affect their willingness to actively participate in collaborative vulnerability disclosure. Thirdly, due to the timeliness of vulnerabilities and the convenience of online transactions, the concealment of security researchers’ illegal behavior is high, greatly increasing the difficulty for the government to detect and punish, which will cause risks to diffuse. To address the existing issues, regulatory mechanisms are necessary.

Regulatory mechanisms refer to the set of processes, methods, and standards through which authorities regulate the participants’ behaviors [3]. Although many scholars have studied regulatory mechanisms, we have the following innovations. First, from the perspective of the subject fields, previous research mostly approached the topic in the legal field, while this paper innovatively takes a management perspective to explore various regulatory mechanisms to standardize stakeholders’ behavior. Second, from the perspective of the research subjects, unlike previous research that focused on regulating ethical hackers, this paper considers the regulation of SRCs and security researchers, particularly exploring how to incentivize them to adopt cooperative strategies while balancing their interests. Third, from the perspective of the application scenarios, previous research on regulatory mechanisms for vulnerability disclosure primarily studied in a traditional context without considering the emerging service of security crowd-testing. It is necessary to explore whether and how traditional regulatory mechanisms adapt to the new issues. Specifically, we delve into scientific questions of what regulatory mechanisms should be adopted by each regulator and how to adjust policies in response to each participant’s reaction to different mechanisms. Last but not least, from the perspective of the research methods, previous research mostly used theoretical frameworks to introduce vague and descriptive security crowd-testing regulatory mechanisms, while this paper draws on the research methodology of regulatory mechanisms in related fields and employs evolutionary game theory to study explicit and quantitative regulatory mechanisms, providing a more intuitive representation of the characteristics and their constraining effects on multiple parties’ behaviors.

Our research objectives and implications are applied to both theory and practice. From a theoretical perspective, firstly, we aim to analyze the vulnerability disclosure behaviors in multi-agent interaction scenarios, delving into the motivations and influencing factors of participants’ strategic choices to gain a more comprehensive understanding of the essence of vulnerability disclosure in security crowd-testing. Secondly, by conducting research into the behavioral evolution process of different participants, including SRCs, security researchers, and governments, we provide new theoretical perspectives for the security crowd-testing field. Furthermore, our research aims to thoroughly analyze the operations and effects of various regulatory mechanisms, providing theoretical support for improving the vulnerability disclosure system in security crowd-testing. From a practical perspective, regulatory mechanisms and their applications can better adapt to the constantly changing vulnerability disclosure environment, enhancing their practical effectiveness. Secondly, by analyzing participants’ strategic responses to changes in different regulatory mechanisms, we assist governments in formulating more adaptable regulatory policies and provide more attractive incentives for SRCs, which effectively guide all participants to adopt more proactive behavior, enhancing the stability of the security crowd-testing industry, and promoting the long-term healthy development of the vulnerability disclosure system.

Section 2 reviews the previous studies on vulnerability disclosure behavior and security crowd-testing. Section 3 proposes a set of assumptions considering practical problems, and constructs the model of the evolutionary game. Based on this, Section 4 presents the stability analysis. Section 5 reveals the dynamic evolution law and how the key parameters impact regulatory mechanisms by numerical simulation. Section 6 summarizes the main conclusions and limitations. In the end, this paper presents future research perspectives.

## 2 Literature review

### 2.1 Vulnerability disclosure behavior

Many scholars have investigated the reasons for the participants’ engagement in vulnerability disclosure behavior from three aspects: participants’ motivation, participants’ characteristics, and trust relationship. First, the participants’ motivation for vulnerability disclosure behavior encompasses both internal and external factors. According to self-determination theory, internal motivation refers to the drive resulting from the participants’ psychological needs, including the need for autonomy, competence, and relatedness [4]. The need for autonomy is manifested by a desire to engage in vulnerability disclosure based on personal interests and beliefs, with interests being a primary driver [5, 6]. The need for competence is expressed as seeking affirmation of one’s abilities during the disclosure process; participants’ enthusiasm increases when they recognize their capability to discover vulnerabilities or contribute to patch management [7]. The need for relatedness involves establishing safe and enjoyable connections with others, such as software vendors forming a “Fixers’ Alliance” or security researchers making connections on cybersecurity forums [8, 9]. External motivation refers to drives from external influences, such as obtaining rewards(money, gifts) [10, 11], or gaining reputations(hall of fame, industry prestige) [5, 7]. It has been shown that participants weigh the expected utility against the expected cost (time, effort), and when the utility is greater than the cost, motivation to participate in vulnerability disclosure is higher [2, 12]. Second, Second, regarding the participants’ characteristics, many scholars have sorted out the characteristics of vulnerability disclosure behavior from the vulnerability life-cycle perspective, i.e. vulnerability discovery, exploitation, and patching respectively. Most of the research findings are focused on vulnerability discovery. For instance, Zhao et al. [8, 13] found that few security researchers can disclose all the vulnerabilities, and the number of vulnerability discoveries they may handle follows a power-law distribution. Votipka et al. [14] demonstrated that experience and knowledge are essential factors influencing participants’ vulnerability disclosure behavior by comparing the vulnerability discovery methods. Maillart et al. [2] argued that the vulnerability disclosure capabilities of the participants decrease exponentially with an increase in the number of vulnerabilities discovered, which means there is a significant productivity gap among the participants, and few of them may discover the vulnerabilities efficiently [15]. In terms of vulnerability exploitation, Canann [16] found that the higher the attack level, the greater the ability of the exploiter, and the greater the spread of the attack. In terms of vulnerability patching, Sen et al. [17] pointed out that vulnerability patchers’ ability, including the time and number of vulnerability patches, is positively correlated with the vulnerability patching rate. Ruohonen et al. [18] argued that most products have vulnerabilities in their early stages of development due to the economics of the software industry, which directly impacts the effectiveness of security researchers’ vulnerability disclosure behavior. Third, trust relationship among participants is a prerequisite for vulnerability disclosure [9]. Zhao et al. [5] found that due to the information asymmetry in the software market, the public faces to uncertain risks of vulnerability disclosure, and security commitments from enterprises to the public incentivize them to engage in vulnerability disclosure. Meanwhile, the transparent and accurate information from enterprises to security researchers helps enhance their trust relationships [19], which makes it possible to institutionalize the ethical hacker culture [20].

As vulnerability disclosure behavior is intricate with diverse strategies adopted by relevant participants, appropriate regulatory mechanisms should be investigated to constrain their behaviors, which has been focused on two main topics: legal boundary and legal risk. Legal boundary is a prerequisite for clarifying the legality of vulnerability disclosure behavior. There is a legal gray area for vulnerability disclosure, where enterprises find it difficult to distinguish the intentions of security researchers. Malicious researchers create risks by exploiting undiscovered vulnerabilities in applications, networks and services [21, 22]. Even ethical researchers could be considered illegal if they accessed or controlled software and hardware without authorization during the process of reporting vulnerabilities [23]. Therefore, a clear legal framework is developed to define security researchers’ behavioral boundaries. The other research hotspot is legal risk, which is a key factor hindering security researchers from engaging in vulnerability disclosure. It showed that legal restrictions are cited as a reason for non-cooperative vulnerability disclosure by 60 percent of security researchers outside the enterprises [3]. Akgul et al. [24] argued that if security researchers’ rights cannot be guaranteed, even though they report vulnerabilities ethically, enterprises may transfer the responsibility of discovering vulnerabilities to them to avoid bearing the costs and liabilities, which causes responsibility dumping. If the rights and responsibilities of vulnerability disclosure are clarified, then security researchers are willing to engage in vulnerability disclosure because it shields them from the risk of legal litigation [25]. However, excessive restrictions or inconsistent legislation might result in a “chilling effect”, decreasing security researchers’ willingness to disclose vulnerabilities [26], which adversely affects the vulnerability disclosure ecosystem [13].

### 2.2 Security crowd-testing

Although regulation of vulnerability disclosure behavior among hackers has been studied for many years, the emergence of new technologies and service models necessitates the collective participation of multiple stakeholders such as enterprises and service companies in collaborative disclosure. Especially, With the increasing prominence of security issues in fields of industry, healthcare, and the Internet of Things (IoT) [27–30], hackers exploiting vulnerabilities in artificial intelligence [31], blockchain [32], and intrusion detection systems [33] to launch large-scale targeted attacks have become norm. The demand for efficient data analysis and processing [34, 35], network security protection [27], and privacy data protection [32] is steadily increasing for enterprises. Security crowd-testing services like Bug Bounty Programs, Vulnerability Reward Programs (VRPs), and Crowdsourcing Software Testing have become crucial means for discovering vulnerabilities in these emerging technologies and ensuring their effective operation.

Some scholars have delved into the effectiveness of disclosing vulnerabilities through security crowd-testing platforms in safeguarding cybersecurity [36–38]. Pascariu et al. [39] argued that security crowd-testing complements enterprises’ cybersecurity management. By offering bounty rewards to vulnerability discoverers and encouraging them to compete with malicious researchers, it is possible to reduce the risk of initial attacks and the probability of vulnerabilities being exploited.

The motivation of security researchers in security crowd-testing has been extensively studied [1, 13]. The findings indicated that money is a significant incentive to discover and disclose vulnerabilities [15, 40, 41]. Finifter et al. [42] found that in Google’s VRP and Mozilla’s Firefox VRP, variable rewards and incentive mechanisms are more attractive to white-hat hackers. Additionally, some security researchers are driven by intrinsic motivation, such as enjoyment and a desire for learning [43]. Meanwhile, due to the heterogeneity among security researchers, social status improvement, knowledge acquisition, or altruism, etc. may become their primary motivations [14]. On the contrary, the mismatch between the abilities of security researchers and the necessary vulnerability discovery skills diminishes their willingness to participate [44], so as the unclear rules and uncertain legal risks in the vulnerability disclosure process or security crowd-testing platforms [45].

The mechanisms to promote active vulnerability disclosure behaviors of security researchers in security crowd-testing have been a recent hotspot. From the perspective of the crowdsourcing platform, Luna [15] found a positive correlation between the completeness of security crowd-testing rules and the willingness of vulnerability disclosure behavior in HackerOne. Ahmed et al. [46] discovered that redundant and ineffective disclosure reports decrease the population of experienced white-hat hackers. From a macro-policy perspective, Zhao et al. [5] evaluated various policies by developing economic models and found that incentive mechanisms are more effective for security researchers. From guiding participant behavior perspective, numerous scholars employed methods such as machine learning, deep learning, and others capable of efficiently identifying and classifying characteristics to monitor data and predict behavior [31, 33], and often using confusion matrices to evaluate their results [47]. These methods often focus on accurate predictions of the behavior of individual participants, while the analysis of regulatory mechanisms typically involves multiple stakeholders including regulators and those being regulated, whose behaviors interact with each other. Therefore, in addressing such issues, game theory has become the most suitable and preferred approach that is applicable for studying behavior interactions and strategy selection. Xiong et al. [48] constructed a game model considering third-party vulnerability-sharing platforms and found that security researchers’ vulnerability disclosure behaviors are encouraged by establishing a credit system for patch development and improving the punishment mechanism for dishonesty. Xu et al. [49] developed a game model to confirm government punishment mechanisms can facilitate win-win situations for enterprises and consumers in certain scenarios. Further, evolutionary game theory has become the preferred choice for most scholars to investigate regulatory mechanisms due to its applicability in studying the dynamic evolution of the long-term behavior of multiple parties. Chen et al. [50] constructed an evolutionary game model of the government and enterprises which explored the constraints of government tax subsidy mechanism on the behavior of the participants. Zhou et al. [51] focused on a punishment mechanism within a reasonable range which is more effective than an incentive mechanism, and proposed suggestions such as intervening as early as possible, and gradually weakening the regulation after stabilizing. Chen et al. [52] also pointed out that a high degree of subsidies will not play a role in restraining the behavior of the main parties, and the government should set up reasonable incentive mechanisms to prevent potential “incentive redundancy”. Chen et al. [53] took a long-term perspective and argued that the enhancement of government reputation plays a crucial role in constraining the behavior of other agents.

It is worth noting that traditional game models require the rational players. However, such strict conditions are not satisfied by the SRCs, security researchers, and the government in reality. For example, security researchers may be irrationally driven by huge profits, hiding their illegal behaviors from SRCs and governments without being detected. Therefore, traditional game models are not suitable in our paper. The evolutionary game model overcomes the above drawbacks and does not require the players to be completely rational. Hence, the evolutionary game model is developed to analyze the stakeholders’ vulnerability disclosure behaviors in the security crowd-testing service.

In summary, the gaps between existing research and this paper are: 1) Existing research primarily has focused on the reasons for participating in vulnerability disclosure and the legal boundaries and risks faced by security researchers, while there is relatively little research on the regulation of vulnerability disclosure behavior. 2) It has been confirmed that security crowd-testing is an effective means to promote vulnerability disclosure, and many scholars have conducted research on the motivation, characteristics, and trust of responders (enterprises) and discoverers (security researchers). Although the interdependence of interests and behaviors among participants has proven to be pervasive, research on their interactions has been limited. 3) Previous research has mainly explored reasons for the low willingness of discoverers (security researchers) to participate in vulnerability disclosure from a legal perspective, but rarely investigated managerial regulatory mechanisms for guiding operators (SRCs) and discoverers (security researchers) to collaborative disclosure. 4) It has been confirmed that incentive mechanisms can encourage security researchers to engage in vulnerability disclosure in security crowd-testing, most studies focus on platforms’ reward mechanisms and the government’s punishment mechanisms. The scope and variety of these mechanisms are relatively limited, leading to a lack of the theoretical foundation for guiding the healthy development of the security crowd-testing industry. Therefore, focusing on promoting collaborative vulnerability disclosure among all parties under security crowd-testing, this paper constructs an evolutionary game model considering factors such as punishments, rewards, trust costs, illegal benefits, etc. We explore the interactions of SRCs, security researchers, and the government to propose regulation mechanisms including reward and punishment mechanisms, trust mechanisms, publicity and training mechanisms, and further analyze the impact of these mechanisms on tripartite parties’ behaviors. This paper aims to provide theoretical support and practical recommendations for improving regulatory mechanisms to facilitate collaborative vulnerability disclosure considering security crowd-testing. The differences between the existing literature and this paper are shown in Table 1.

**Table 1:** Differences between the existing literature and this paper.

Perspective	Previous Research		Proposed work	Differentiation
	Pros	Cons		
**Research Focus**	Proved the existence of multiple participants and interactions	Individual participant strategy	Multiple participants strategies	Strategies under multi-participant interaction
**Research Scope**	Analyzed the relevant legal policies	The reason for participants’ low willingness	Mechanisms to regulate participants’ behaviors	Behavior guidance
**Types of regulatory mechanisms**	Proved the incentive mechanisms’ effectiveness	Reward and punishment mechanisms	Reward mechanisms, punishment mechanisms, trust mechanisms, publicity and training mechanisms	Implications of multiple regulatory mechanisms
**Scenarios of regulatory mechanisms**	Proposed mechanisms for regulators	Government regulates security researchers	Government regulates SRCs, government regulates security researchers, SRCs’ regulate security researchers, etc	Application of multiple regulatory scenarios

## 3 Model formulation

### 3.1 Problem description

In the security crowd-testing process, SRCs need to communicate directly with security researchers and be regulated by the government as well. Hence, there are three players in the game including SRCs, security researchers, and the government.

Generally, SRCs are established by technically proficient enterprises, attracting security researchers to report vulnerabilities in a platform. Although the costs are relatively high, forming a collaborative vulnerability reporting system with security researchers outside the enterprises can enhance their abilities to deal with vulnerabilities, which simplifies the vulnerability disclosure process. However, during the operation of SRCs, their mismanagement may conflict with security researchers, such as inconsistencies in vulnerability rating rules, disputes over the methods of vulnerability testing, and unclear vulnerability reward mechanisms, etc. In this situation, SRCs usually employ two strategies, i.e., “active management” and “negative management”.

Security researchers are providers of security crowd-testing services, mainly comprising internal experts from enterprises and external white-hat hackers from the hacker community. Due to the wide variety of security researchers, it is challenging to detect and restrict their behaviors effectively. Plus, their motivations are various, including personal interests, beliefs, and self-affirmation. But most of them are primarily driven by external factors such as rewards (money, gifts) and reputation (hall of fame, industry prestige), etc. When profits are significant, security researchers may take illegal behaviors that violate crowd-testing rules, and they may even sell discovered vulnerability information to the black market. Therefore, security researchers have two strategies, i.e., “legal participation” and “illegal participation”.

The government primarily refers to agencies responsible for cybersecurity regulation, including government departments such as the Cyber Security Office, National Security Agency, Ministry of Industry and Information Technology, as well as cybersecurity technology centers such as Computer Emergency Response Teams and Center for Internet Security. These agencies are responsible for managing vulnerability disclosure activities, and their responsibilities encompass coordinating the vulnerability disclosure process, promoting collaborative vulnerability disclosure and real-time sharing of vulnerability information, jointly assessing and managing vulnerability risks, and against illegal activities related to vulnerability disclosure. Theoretically, both SRCs and security researchers are regulated by the government. However, in reality, vulnerability disclosure spans various industries with numerous entities. In the condition of limited personnel, financial, and material resources, government regulatory efforts may vary significantly. Therefore, the government typically adopts two behavioral strategies, i.e., “strict regulation” and “lax regulation”.

**Fig 2:**
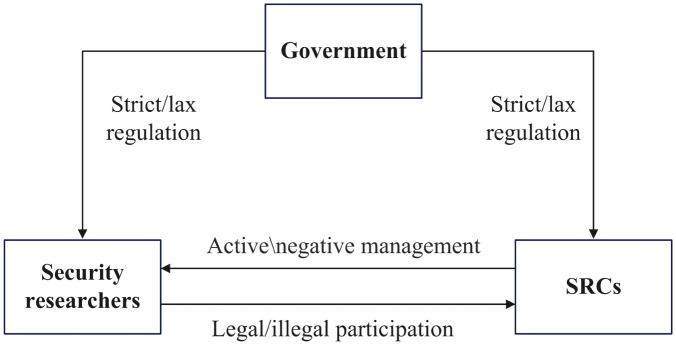
Game relationship among SRCs, security researchers and the government.

In cases where SRCs are driven by negative management motivations stemming from factors like time, cost, and funding, it may result in inadequate management, a lack of respect for the security researchers’ efforts, and a deficit in effective communication with them. This may lead to mistrust between SRCs and security researchers, which further affects their cooperative relationship, causing losses for both parties. Security researchers help SRCs discover vulnerabilities but are also driven by their own interests, so they do not always prioritize SRCs’ rules. When security researchers are legal participants, they receive bounties from SRCs based on the severity and value of the vulnerabilities. However, when they are illegal participants, they may violate SRCs’ rules and face punishments under SRCs’ positive management, while they also could gain illegal benefits. For the government, it punishes SRCs and security researchers who violate legal regulations, while also rewarding those SRCs who engage in positive management. The game relationship among these three parties is shown in Fig 2.

### 3.2 Assumptions and variables

**Assumption 1**. *The set of strategies for SRCs is {active management, negative management}, the probability of active management is* *x*, *and the probability of negative management is* 1 − *x; the set of strategies for security researchers is {legal participation, illegal participation}, the probability of legal participation is* *y, and the probability of illegal participation is* 1 − *y; the set of strategies for the government is {strict regulation, lax regulation}, the probability of strict regulation is z, and the probability of lax regulation is* 1 − *z, in which* *x*, *y and z* ∈ [0, 1].

**Assumption 2**. *The basic benefits for SRCs participating in security crowd-testing are* *S*_1_, *which include obtaining vulnerability information to improve system security, enhancing user trust, and establishing a positive credit of actively addressing security issues. The basic costs of SRCs are C*_1_, *including operational costs and incentive costs. Operational costs cover expenses for running and maintaining the security crowd-testing platform, formulating vulnerability crowd-testing guidelines, auditing vulnerability quality, and assessing vulnerability risks. Incentive costs include expenses for organizing cybersecurity skills competitions, vulnerability bounties, team bonuses, credit rewards, honor lists, etc. It is worth noting that there is a superlinear relationship between the number of security researchers and the number of vulnerabilities discovered. In other words, the more security researchers participate, the higher the quality of SRCs’ services and the system’s security level. In the case of positive management, a higher degree of active management α will attract more security researchers, resulting in higher benefits and costs. Therefore, the benefits of SRCs’ active management are αS*_1_, *and the costs are αC*_1_. *Additionally, SRCs’ negative management receives additional benefits S*_2_, *for example, concealing the authenticity and severity of vulnerabilities to reduce the bounties paid to security researchers, where S*_1_ + *S*_2_ > *αS*_1_. *Security researchers of illegal participation cause losses to SRC’ L*_1_, *including vulnerability information leakage and exploitation of vulnerabilities*.

**Assumption 3**. *The costs of security researchers to participate in security crowd-testing are C*_2_, *including tools, time, and effort required to discover vulnerabilities, which are the same for legal and illegal participation. The benefits for security researchers’ legal participants are P*_1_, *which include bounties, reputation, and credits earned, while those who illegally participate earn higher benefits of P*_2_, *including benefits from infiltrating other systems or illegally selling vulnerabilities, in which P*_1_ < *P*_2_. *SRCs with positive management can detect the illegal behaviors in time and impose punishments of F, such as freezing credit rewards, banning the conversion of bonuses, etc. However, in the case of negative management, SRCs may not detect security researchers’ illegal behaviors promptly. Additionally, SRCs’ negative management causes losses of L*_2_ *to security researchers who participate legally, such as not responding promptly or refusing to acknowledge vulnerabilities submitted by researchers, in which L*_2_ ≤ *P*_1_ < *P*_2_.

**Assumption 4**. *The costs of the government’s strict regulation are C*_3_, *which include costs of improving the establishment of a supervisory system for vulnerability disclosure, inspections of non-compliance with disclosure regulations, optimization of cybersecurity vulnerability management technologies, and participation in vulnerability disclosure audit. Strict regulation can increase public satisfaction and enhance government credibility, resulting in regulatory benefits for the government of R. To promote collaborative vulnerability disclosure, the government usually implements rewards and punishments measures. Specifically, the government provides rewards of A for SRCs’ active management, imposes punishments of K*_1_ *for SRCs’ negative management, and gives punishments of K*_2_ *for security researchers of illegal participation, such as warnings, fines and rectifications. In the case of lax regulation, there are no regulatory costs or benefits*.

**Assumption 5**. *Trust between SRCs and security researchers is crucial. In the case of active management, SRCs always pay additional trust costs C*_4_ *to establish and maintain long-term effective trust relationships, including the establishment of vulnerability reporting response mechanisms, timely coordination and communication, feedback on the progress of vulnerability disclosure, etc. At this time, the higher level of trust between the two parties brings trust benefits of S*_3_ *to SRCs. For example, security researchers tend to participate in crowd-testing tasks from SRCs with higher trustworthiness. In contrast, SRC’ negative management are unwilling to pay additional trust costs, leading to an escalation of conflicts, resulting in losses of L*_3_ *to SRCs, such as the loss of security researchers boycotting SRCs’ crowd-testing tasks and reputational damage from media coverage, etc. As there is no trust issue between security researchers of illegal participation and SRCs, we do not consider this situation in the game*.

**Assumption 6**. *When SRCs choose active management and security researchers choose legal participation, it brings social benefits of M. However, when SRCs engage in negative management or security researchers engage in illegal participation, the social losses would be W*.

Table 2 presents the parameters along with their meanings.

**Table 2:** The main parameters of the tripartite regulatory game model under security crowd-testing.

Participants	Parameters	Meanings
**SRCs**	*α*	The degree of SRCs’ active management;
	*C*_1_	The basic costs of SRCs;
	*C*_4_	The trust costs of SRCs’ active management;
	*S*_1_	The basic benefits of SRCs;
	*S*_2_	The additional benefits of SRCs’ negative management;
	*S*_3_	The trust benefits of SRCs’ active management;
	*L*_1_	The losses of SRCs caused by security researchers’ illegal participation;
	*L*_3_	The trust losses of SRCs’ negative management;
**Security researchers**	*P*_1_	The benefits of security researchers’ legal participation;
	*P*_2_	The benefits of security researchers’ illegal participation;
	*C*_2_	The benefits of security researchers;
	*F*	The SRCs’ punishments for security researchers’ illegal participation;
	*L*_2_	The losses of security researchers caused by SRCs’ negative management;
**Government**	*R*	The benefits of the government’s strict regulation;
	*C*_3_	The costs of the government’s strict regulation;
	*A*	The government’s rewards for SRCs’ active management;
	*K*_1_	The government’s punishments for SRCs’ negative management;
	*K*_2_	The government’s punishments for security researchers’ illegal participation;
	*M*	Social welfare when SRCs choose active management and security researchers choose illegal participation;
	*W*	Social losses caused by SRCs’ negative management or security researchers’ illegal participation.

### 3.3 Model construction

Based on the assumptions and parameters defined, the game payoff matrix of the tripartite is shown in Table 3.

**Table 3:** Payment matrix of evolution game among SRCs, security researchers and the government.

Strategies	SRCs	Security researchers	Government
(*x*,*y*,*z*)	*αS*_1_ + *S*_3_ + *A* − *αC*_1_ − *C*_4_	*P*_1_ − *C*_2_	*R* + *M* − *C*_3_ − *A*
(*x*,*y*,1 − *z*)	*αS*_1_ + *S*_3_ − *αC*_1_ − *C*_4_	*P*_1_ − *C*_2_	*M*
(*x*,1 − *y*,*z*)	*αS*_1_ + *A* − *αC*_1_ − *C*_4_ − *L*_1_ + *F*	*P*_1_ − *C*_2_ − *K*_2_ − *F*	*R* + *K*_2_ − *C*_3_ − *A* − *W*
(*x*,1 − *y*,1 − *z*)	*αS*_1_ − *αC*_1_ − *C*_4_ − *L*_1_ + *F*	*P*_2_ − *C*_2_ − *F*	−*W*
(1 − *x*,*y*,*z*)	*S*_1_ + *S*_2_ − *K*_1_ − *C*_1_ − *L*_3_	*P*_1_ − *C*_2_ − *L*_2_	*R* + *K*_1_ − *C*_3_ − *W*
(1 − *x*,*y*,1 − *z*)	*S*_1_ + *S*_2_ − *C*_1_ − *L*_3_	*P*_1_ − *C*_2_ − *L*_2_	−*W*
(1 − *x*,1 − *y*,*z*)	*S*_1_ + *S*_2_ − *K*_1_ − *C*_1_ − *L*_1_	*P*_2_ − *C*_2_ − *K*_2_	*R* + *K*_1_ + *K*_2_ − *C*_3_ − *W*
(1 − *x*,1 − *y*,1 − *z*)	*S*_1_ + *S*_2_ − *C*_1_ − *L*_1_	*P*_2_ − *C*_2_	−*W*

## 4 Evolutionary stability analysis

### 4.1 Stability analysis of SRCs

According to Table 2, the expected income *E*_11_ or *E*_12_ of SRCs when they choose the “active management” or “negative management” strategy is respectively:

$$\begin{array}{c} \begin{array}{cc} & E_{11}=yz(\alpha S_{1}+S_{3}+A-\alpha C_{1}-C_{4})+y\left(1-z\right)(\alpha S_{1}+S_{3}-\alpha C_{1}-C_{4})+\\ & \left(1-y\right)z(\alpha S_{1}+A-\alpha C_{1}-C_{4}-L_{1}+F)+\\ & \left(1-y\right)\left(1-z\right)(\alpha S_{1}-\alpha C_{1}-C_{4}-L_{1}+F) \end{array} \end{array}$$ (1)

$$\begin{array}{c} \begin{array}{cc} & E_{12}=yz(S_{1}+S_{2}-K_{1}-C_{1}-L_{3})+y\left(1-z\right)(S_{1}+S_{2}-C_{1}-L_{3})+\\ & \left(1-y\right)z(S_{1}+S_{2}-K_{1}-C_{1}-L_{1})+\left(1-y\right)\left(1-z\right)(S_{1}+S_{2}-C_{1}-L_{1}) \end{array} \end{array}$$ (2)

The average expected income $\bar{E_{1}}$ of SRCs is:

$$\begin{array}{c} \bar{E_{1}}=xE_{11}+\left(1-x\right)E_{12} \end{array}$$ (3)

According to Formulas 1, 2 and 3, we can further obtain the replicator dynamics equation of SRCs’ strategy as follows:

$$\begin{array}{cc} F(x) & =\frac{dx}{dt}=x\left(E_{11}-{\bar{E}}_{1}\right)=x\left(x-1\right)\\ & \left[S_{1}+S_{2}+\alpha C_{1}+C_{4}-C_{1}-\alpha S_{1}-F+\left(F-L_{3}-S_{3}\right)y-\left(A+K_{1}\right)z\right] \end{array}$$ (4)

The first-order derivatives of *x* and *G*(*y*) are as follows:

$$\begin{array}{c} \frac{d(F(x))}{dx}=\left(2x-1\right)[S_{1}+S_{2}+\alpha C_{1}+C_{4}-C_{1}-\alpha \;S_{1}-F-\\ \left(F-L_{3}-S_{3}\right)y-\left(A+K_{1}\right)z] \end{array}$$ (5)

$$\begin{array}{c} G\left(y\right)=S_{1}+S_{2}+\alpha C_{1}+C_{4}-C_{1}-\alpha \;S_{1}-F+(F-L_{3}-S_{3})y-(A+K_{1})z \end{array}$$ (6)

In order to find the probability of SRCs choosing active management in the steady state, it must be satisfied that *F*(*x*) = 0, and $\frac{d(F(x))}{dx}<0$. As ∂*G*(*y*)/∂*y* < 0, *G*(*y*) is a decreasing function with respect to *y*.

**Fig 3:**
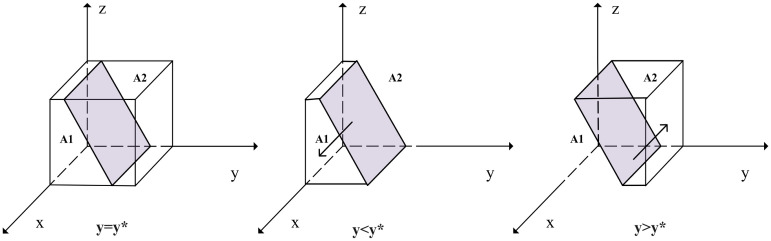
Strategy evolution phase diagram of SRCs.

When *y* = *S*_1_ + *S*_2_ + *αC*_1_ + *C*_4_ − *C*_1_ − *αS*_1_ − *F* − (*A* + *K*_1_) *z*/*L*_3_ + *S*_3_ − *F* = y**, *G*(*y*) = 0, so $\frac{d(F(x))}{dx}=0$, that is *F*(*x*) = 0, at this time all *x* is in a stable state. When *y* < y*, *G*(*y*) < 0, and *d*(*F*(*x*))/*dx*|_*x* = 0_ < 0, at this time for any *x* = 0 as an evolutionary stabilization strategy for SRCs. Conversely, *x* = 1 is an evolutionary stabilization strategy for SRCs. The strategy evolution phase diagram of SRCs is shown in Fig 3:

Fig 3 shows that the volume of the probability that SRCs choose negative management is $V_{A_{1}}$ of *A*_1_, and the volume of the probability that they choose compliance disclosure behavior is $V_{A_{2}}$ of *A*_2_:

$$\begin{array}{cc} V_{A_{1}} & =\int_{0}^{1}\int_{0}^{1}\frac{S_{1}+S_{2}+\alpha C_{1}+C_{4}-C_{1}-\alpha \;S_{1}-F-(A+K_{1})z}{L_{3}+S_{3}-F}dzdx\\ & =\frac{2S_{1}+2\;S_{2}+2\alpha C_{1}+2C_{4}-2C_{1}-2\alpha \;S_{1}-2F-A-K_{1}}{2(L_{3}+S_{3}-F)} \end{array}$$ (7)

$$\begin{array}{c} V_{A_{2}}=1-V_{A_{1}}=\frac{2L_{3}+2S_{3}+2C_{1}+A+K_{1}-2(S_{1}+S_{2})-2(\alpha C_{1}+C_{4})}{2(L_{3}+S_{3}-F)} \end{array}$$ (8)

**Proposition 1**. *The probability that SRCs choose active management is positively correlated with trust losses and gains, and the governments’ rewards and punishments, while negatively correlated with the benefits of negative management and the costs of active management*.

*Proof*. The probability of SRCs active management is $V_{A_{1}}$, By solving for the first-order partial derivatives of the elements, we get:$\partial V_{A_{1}}/\partial (L_{3}+S_{3})>0$, $\partial V_{A_{1}}/\partial (A+K_{1})>0$, $\partial V_{A_{1}}/\partial (S_{1}+S_{2})<0$, $\partial V_{A_{1}}/\partial (\alpha C_{1}+C_{4})<0$. Therefore, the increase of *L*_3_, *S*_3_, *A*, and *K*_1_, and the decrease of *S*_1_ + *S*_2_ and *αC*_1_ + *C*_4_ can make the SRCs increase the probability of active management.

Proposition 1 indicates that increasing the benefits of SRCs’ active management can reduce the probability of their negative management. Therefore, the government can take various measures to enhance SRCs’ willingness to choose active management, which includes strengthening reward mechanisms and guiding the behavior of SRCs and security researchers through media promotion and policy documents to promote the establishment of the trust relationship. Simultaneously, enhancing the degree of punishments, which can rigorously control the benefits of negative management to decrease the costs of active management, can promote the stable development of SRCs.

### 4.2 Stability analysis of security researchers

According to Table 2, the expected income *E*_21_ or *E*_22_ of security researchers when they choose the “legal participation” or “illegal participation” strategy is respectively:

$$\begin{array}{c} \begin{array}{cc} E_{21}= & \text{xz}(P_{1}-C_{2})+x\left(1-z\right)(P_{1}-C_{2})+\left(1-x\right)z(P_{1}-C_{2}-L_{2})+\\ & \left(1-x\right)\left(1-z\right)(P_{1}-C_{2}-L_{2}) \end{array} \end{array}$$ (9)

$$\begin{array}{c} \begin{array}{cc} E_{22}= & \text{xz}(P_{2}-C_{2}-K_{2}-F)+x\left(1-z\right)(P_{2}-C_{2}-F)+\\ & \left(1-x\right)z(P_{2}-C_{2}-K_{2})+\left(1-x\right)\left(1-z\right)(P_{2}-C_{2}) \end{array} \end{array}$$ (10)

The average expected income $\bar{E_{2}}$ of security researchers is:

$$\begin{array}{c} \bar{E_{2}}=yE_{21}+\left(1-y\right)E_{22} \end{array}$$ (11)

According to Formulas 9, 10 and 11, we can further obtain the replicator dynamics equation of the strategy selection of security researchers as follows:

$$\begin{array}{c} F\left(y\right)=dy/dt=y(E_{21}-\bar{E_{2}})=y\left(y-1\right)[L_{2}+P_{2}-P_{1}-(F+L_{2})x-K_{2}z] \end{array}$$ (12)

The first-order derivative of *y* is as follows:

$$\begin{array}{c} \frac{d(F(y))}{dy}=\left(2y-1\right)[[L_{2}+P_{2}-P_{1}-(F+L_{2})x-K_{2}z] \end{array}$$ (13)

Let:

$$\begin{array}{c} J\left(z\right)=L_{2}+P_{2}-P_{1}-(F+L_{2})x-K_{2}z \end{array}$$ (14)

In order to find the probability of security researchers choosing legal participation in the steady state, it must be satisfied that *F*(*y*) = 0 and *d*(*F*(*y*))/*dy* < 0, which results in *J*(*z*) being a decreasing function.

**Fig 4:**
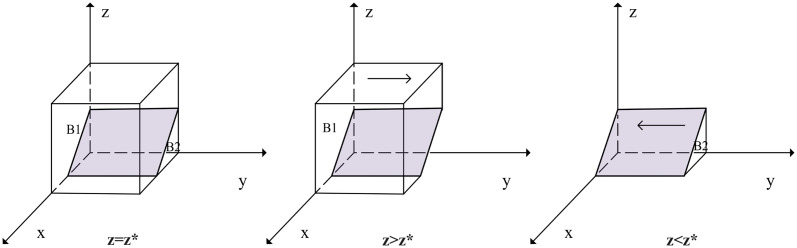
Strategy evolution phase diagram of security researchers.

When *z* = *L*_2_ + *P*_2_ − *P*_1_ − (*F* + *L*_2_) *x*/*K*_2_ = z*, *J*(*z*) = 0, at this time $\frac{d(F(y))}{dy}=0$, for any *y* is in a stable state. When *z* < z*, *G*(*z*) > 0, at this time *d*(*F*(*y*))/*dy*|_*y*=0_ < 0, *y* = 0 is an evolutionary stabilization strategy for security researchers. Conversely, *y* = 1 is an evolutionary stabilization strategy. The strategy evolution phase diagram of security researchers is shown in Fig 4.

According to Fig 4, the volume of the probability of security researchers’ legal participation is $V_{B_{1}}$ of *B*_1_, and the volume of the probability of illegal participation is $V_{B_{2}}$ of *B*_2_:

$$\begin{array}{c} V_{B_{2}}=\int_{0}^{1}\int_{0}^{1}\frac{L_{2}+P_{2}-P_{1}-(F+L_{2})x}{K_{2}}dxdy=\frac{L_{2}+2P_{2}-2P_{1}-F}{2K_{2}} \end{array}$$ (15)

$$\begin{array}{c} V_{B_{1}}=1-V_{B_{2}}=\frac{2K_{2}-L_{2}-2P_{2}+2P_{1}+F}{2K_{2}} \end{array}$$ (16)

**Proposition 2**. *The probability of security researchers’ legal participation is positively correlated with the benefits of legal participation and the punishments imposed by the government and SRCs, while negatively correlated with the losses from negative management by SRCs and the benefits of illegal participation*.

*Proof.* Based on the expression for the probability of security researchers’ legal participation $V_{B_{1}}$, the first-order partial derivative of each element is obtained: $\partial V_{B_{1}}/\partial P_{1}>0$, $\partial V_{B_{1}}/\partial K_{2}>0$, $\partial V_{B_{1}}/\partial F>0$, $\partial V_{B_{1}}/\partial L_{2}<0$, $\partial V_{B_{1}}/\partial P_{2}<0$. Thus, both an increase in *P*_1_, *K*_2_, and *F* and a decrease in *P*_2_ and *L*_2_ both increase the probability of security researchers’ legal participation.

Proposition 2 suggests that when the illegal benefits of security researchers participating illegally are too high, the government should strengthen regulation. Additionally, the government can reduce the probability of security researchers’ illegal participation by cooperating with SRCs in regulation and providing timely punishments.

### 4.3 Stability analysis of the government

Similarly, the expected income *E*_31_ or *E*_32_ of the government when choosing “strict regulation” or “lax regulation” strategy is respectively:

$$\begin{array}{c} \begin{array}{cc} E_{31}= & xy(R+M-C_{3}-A)+x\left(1-y\right)(R+K_{2}-C_{3}-A-W)\\ & +\left(1-x\right)y(R+K_{1}-C_{3}-W)+\left(1-x\right)\left(1-y\right)(R+K_{1}+K_{2}-C_{3}-W) \end{array} \end{array}$$ (17)

$$\begin{array}{c} E_{32}=xyM+x\left(1-y\right)\left(-W\right)+\left(1-x\right)y\left(-W\right)+\left(1-x\right)\left(1-y\right)\left(-W\right) \end{array}$$ (18)

The average expected income $\bar{E_{3}}$ of the government is:

$$\begin{array}{c} \bar{E_{3}}=zE_{31}+\left(1-z\right)E_{32} \end{array}$$ (19)

According to Formulas 17, 18 and 19, we can further obtain the replicator dynamics equation of the behavior strategy selection of the government as follows:

$$\begin{array}{c} F\left(z\right)=dz/dt=z(E_{31}-\bar{E_{3}})=z\left(z-1\right)[C_{3}-K_{1}-K_{2}-R+(A+K_{1})x+K_{2}y] \end{array}$$ (20)

The first-order derivatives of *z*, and the set *H*(*y*) are as follows:

$$\begin{array}{c} \frac{d(F(z))}{dz}=\left(2z-1\right)[C_{3}-K_{1}-K_{2}-R+(A+K_{1})x+K_{2}y] \end{array}$$ (21)

$$\begin{array}{c} H\left(y\right)=C_{3}-K_{1}-K_{2}-R+(A+K_{1})x+K_{2}y \end{array}$$ (22)

The government chooses strict regulation in a steady state must be satisfied that *F*(*z*) = 0, and *d*(*F*(*z*))/*dz* < 0. It can be derived that ∂*H*(*y*)/∂*y* > 0, *H*(*y*) is an increasing function with respect to *y*.

**Fig 5:**
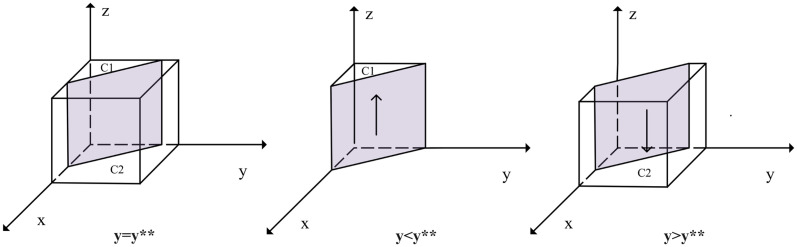
Strategy evolution phase diagram of the government.

When *y* = *C*_3_ − *K*_1_ − *K*_2_ − *R* + (*A* + *K*_1_) *x*/*K*_2_ = y**, at this time *H*(*y*) = 0, and $\frac{d(F(z))}{dz}=0$, for any *z* is in a stable state. When *y* < y*, *G*(*z*)>0, at this time *H*(*y*) < 0, and *d*(*F*(*z*))/*dz*|_*z*=1_ > 0, *z* = 1 is an evolutionary stabilization strategy. Conversely, *z* = 0 is an evolutionary stabilization strategy. The strategy evolution phase diagram of the government is shown in Fig 5.

From Fig 5, the volume of the probability that the government strict regulation is $V_{C_{1}}$ of *C*_1_, and the volume of the probability that the government strict regulation is $V_{C_{2}}$ of *C*_2_:

$$\begin{array}{c} V_{C_{1}}=\int_{0}^{1}\int_{0}^{1}\frac{C_{3}-K_{1}-K_{2}-R+(A+K_{1})x}{K_{2}}dxdz=\frac{2C_{3}-K_{1}-2K_{2}-2R+A}{2K_{2}} \end{array}$$ (23)

$$\begin{array}{c} V_{C_{2}}=1-\frac{2C_{3}-K_{1}-2K_{2}-2R+A}{2K_{2}}=\frac{K_{1}-2C_{3}+6K_{2}+2R-A}{2K_{2}} \end{array}$$ (24)

**Proposition 3**. *The probability that the government implements strict regulation is positively correlated with its punishments for SRCs and security researchers, and regulatory benefits, while negatively correlated with the costs of strict regulation and rewards of the government*.

*Proof*. According to the $V_{C_{1}}$, it is derived that $\partial V_{C_{1}}/\partial K_{1}>0$, $\partial V_{C_{1}}/\partial K_{2}>0$, $\partial V_{C_{1}}/\partial R>0$, $\partial V_{C_{1}}/\partial C_{3}<0$, $\partial V_{C_{1}}/\partial A<0$. Therefore, an increase in *K*_1_, *K*_2_, *R* and a decrease in *C*_3_, *A* can lead to an increase in the probability of the government’s strict regulation.

Proposition 3 indicates that the severity of government punishments is positively correlated with the degree of strict regulation and negatively correlated with the level of rewards. In other words, higher regulatory benefits can incentivize the government to rigorously fulfill its regulatory responsibilities.

### 4.4 Systematic equilibrium point analysis of the tripartite evolutionary game

The above equilibrium point is not completely an evolutionary stability strategy for replicating a dynamic system. In asymmetric games, the mixed strategy equilibrium points are saddle points, and the strategies at strict Nash equilibrium are all pure. Therefore, we only focus on analyzing the stability of the eight pure strategy equilibrium points. It is necessary to further discuss the stability of the system equilibrium point by using the Jacobian matrix local stability analysis method. According to the Lyapunov theorem, when all the eigenvalues in the Jacobi matrix are satisfied with negative real parts, then the potential equilibrium point represents an evolutionarily stable strategy in the evolutionary game. The Jacobian matrix of the tripartite evolutionary game system is

$$\begin{array}{c} J=[\begin{array}{ccc} J_{1} & J_{2} & J_{3}\\ J_{4} & J_{5} & J_{6}\\ J_{7} & J_{8} & J_{9} \end{array}]=[\begin{array}{ccc} \partial F(x)/\partial x & \partial F(x)/\partial y & \partial F(x)/\partial z\\ \partial F(y)/\partial x & \partial F(y)/\partial y & \partial F(y)/\partial z\\ \partial F(z)/\partial x & \partial F(z)/\partial y & \partial F(z)/\partial z \end{array}] \end{array}$$ (25)

Where

$$\begin{array}{c} J_{1}=\left(2x-1\right)[S_{1}+S_{2}+\alpha C_{1}+C_{4}-C_{1}-\alpha \;S_{1}-F+(F-L_{3}-S_{3})y-(A+K_{1})z] \end{array}$$ (26)

$$\begin{array}{c} J_{2}=x\left(x-1\right)(F-L_{3}-S_{3}) \end{array}$$ (27)

$$\begin{array}{c} J_{3}=x\left(x-1\right)(-A-K_{1}) \end{array}$$ (28)

$$\begin{array}{c} J_{4}=y\left(y-1\right)(-F-L_{2}) \end{array}$$ (29)

$$\begin{array}{c} J_{5}=\left(2y-1\right)[L_{2}+P_{2}-P_{1}-(F+L_{2})x-K_{2}z] \end{array}$$ (30)

$$\begin{array}{c} J_{6}=y\left(y-1\right)(-K_{2}) \end{array}$$ (31)

$$\begin{array}{c} J_{7}=z\left(z-1\right)(A+K_{1}) \end{array}$$ (32)

$$\begin{array}{c} J_{8}=z\left(z-1\right)K_{2} \end{array}$$ (33)

$$\begin{array}{c} J_{9}=\left(2z-1\right)[C_{3}-K_{1}-K_{2}-R+(A+K_{1})x+K_{2}y] \end{array}$$ (34)

The eigenvalues of the Jacobian matrix corresponding to the eight equilibrium points and the system stability are shown in Table 4.

**Table 4:** Eigenvalues corresponding to pure strategy equilibrium points.

Equilibrium Point	Eigenvalue			Stability
	λ_1_	λ_2_	λ_3_	
*E*_1_(0, 0, 0)	C_1_ + *αS*_1_ + *F* − *S*_1_ − *S*_2_ − *αCl* − C_4_ (−)	*P*_1_ − *L*_2_ − *P*_2_ (−)	K_1_ − *C*_3_ + *K*_2_ + *R* (−)	Stable point
*E*_2_(0, 1, 0)	*C*_1_ + *L*_3_ + *S*_3_ + *αS*_1_ − *C*_4_ − *S*_1_ − *S*_2_ − *αC*_1_ (−)	*L*_2_ − *P*_1_ + *P*_2_ (+)	*K*_1_ − *C*_3_ + *R* (+)	Unstable point
*E*_3_(0, 0, 1)	*A* + *C*_1_ + *K*_1_ + *F* + *αS*_1_ − *C*_4_ − *S*_1_ − *S*_2_ − *αC*_1_ (−)	*K*_2_ + *P*_1_ − *P*_2_ − *L*_2_ (−)	*C*_3_ − *K*_1_ − *K*_2_ − *R* (−)	Stable point
*E*_4_(0, 1, 1)	*A* + *C*_1_ + *K*_1_ + *L*_3_ + *S*_3_ + *αS*_1_ − *C*_4_ − *S*_1_ − *S*_2_ − *αC*_1_ (−)	*L*_2_ + *P*_2_ − *P*_1_ − *K*_2_ (−)	*C*_3_ − *K*_1_ − *R* (−)	Stable point
*E*_5_(1, 0, 0)	*S*_1_ + *S*_2_ + *αC*_1_ + *C*_4_ − *αS*_1_ − *C*_1_ − *F* (−)	*F* + *P*_1_ − *P*_2_ (+)	*K*_2_ − *C*_3_ − *A* + *R* (+)	Unstable point
*E*_6_(1, 1, 0)	*C*_4_ + *S*_1_ + *S*_2_ + *αC*_1_ − *S*_3_ − *C*_1_ − *L*_3_ − *αS*_1_ (−)	*P*_2_ − *P*_1_ − *F* (−)	*R* − *C*_3_ − *A* (−)	Stable point
*E*_7_(1, 0, 1)	*C*_4_ + *S*_1_ + *S*_2_ + *αC*_1_ − *C*_1_ − *F* − *K*_1_ − *αS*_1_ − *A* (−)	*F* + *K*_2_ + *P*_1_ − *P*_2_ (−)	*A* + *C*_3_ − *K*_2_ − *R* (−)	Stable point
*E*_8_(1, 1, 1)	*C*_4_ + *S*_1_ + *S*_2_ + *αC*_1_ − *C*_1_ − *A* − *K*_1_ − *S*_3_ − *L*_3_ − *αS*_1_ (−)	*P*_2_ − *K*_2_ − *P*_1_ − *F* (−)	*A* + *C*_3_ − *R* (−)	Stable point

It can be seen that *E*_2_(0, 1, 0) is never an equilibrium under any case, indicating the absence of security researchers choosing the legal participation strategy without external incentives. This indirectly illustrates the crucial role played by the government in guiding security researchers’ behavior. As for point *E*_5_(1, 0, 0), when SRCs choose active management, in reality, offering substantial rewards and diverse incentives, security researchers tend to legal participation rather than engage in illegal activities like trading in the black market for vulnerabilities. This point contradicts reality, so it is excluded.

Furthermore, it is calculated that *E*_8_(1, 1, 1) is in a stable state. In this scenario, the government regulation tends to become routine, with SRCs actively communicating and collaborating with it. At this time, SRCs stay updated on the latest regulatory developments, enhance risk management and preventive measures, and avoid non-compliant disclosure behavior. Meanwhile, SRCs make efforts to improve the rules of security crowd-testing, define internal responsibilities, specify the scope of security researchers’ authority, rigorously follow vulnerability approval and authorization procedures, and enhance relevant incentive mechanisms. In this context, the advantages of SRCs’ active management become evident, and they tend to choose active management strategy, i.e., *C*_4_ + *S*_1_ + *S*_2_ + *αC*_1_ − *C*_1_ − *A* − *K*_1_ − *S*_3_ − *L*_3_ − *αS*_1_ < 0. Through the continuous development of SRCs, the legitimacy, security, and stability of the security researcher crowd-testing environment have improved, making it more attractive to security researchers, with *P*_2_ − *K*_2_ − *P*_1_ − *F* < 0, prompting security researchers to prefer the legal participation strategy. For the government, SRCs and security researchers actively cooperate, gradually reducing the government’s regulatory costs, and significantly increasing regulatory benefits, i.e., *A* + *C*_3_ − *R* < 0, leading the government to implement strict regulatory strategy. Ultimately, the system achieves the ideal state of active management, legal participation, strict regulation in collaborative vulnerability disclosure.

## 5 Numerical simulation

To verify the validity of evolutionary stability and the dynamic evolution process of three parties’ collaborative disclosure in security crowd-testing, this paper conducts numerical simulations by MATLAB. Based on the model analysis, the conditions that need to be satisfied: *C*_4_ + *S*_1_ + *S*_2_ + *αC*_1_ − *C*_1_ − *A* − *K*_1_ − *S*_3_ − *L*_3_ − *αS*_1_ < 0, *P*_2_ − *K*_2_ − *P*_1_ − *F* < 0. With reference to the parameter setting method of Liu et al. [54], the parameter values in this study are mainly determined by two methods. Firstly, based on real cases and literature references, we refer to parameter values and research results from Walshe et al. [55] and Zhao et al. [56], setting: *P*_1_ = 20, *P*_2_ = 50, *C*_2_ = 20, *L*_1_ = 60, *L*_3_ = 100. Based on the policy text analysis of the “Cyber Security Law of the People’s Republic of China”, setting: *R* = 100, *C*_3_ = 50, *A* = 20, *K*_1_ = 15, *K*_2_ = 35. Secondly, according to official data from HackerOne and the equilibrium above condition requirements, setting: *α* = 1.5, *C*_1_ = 30, *C*_4_ = 10, *S*_1_ = 90, *S*_2_ = 60, *S*_3_ = 30, *F* = 5, *L*_2_ = 50.

**Fig 6:**
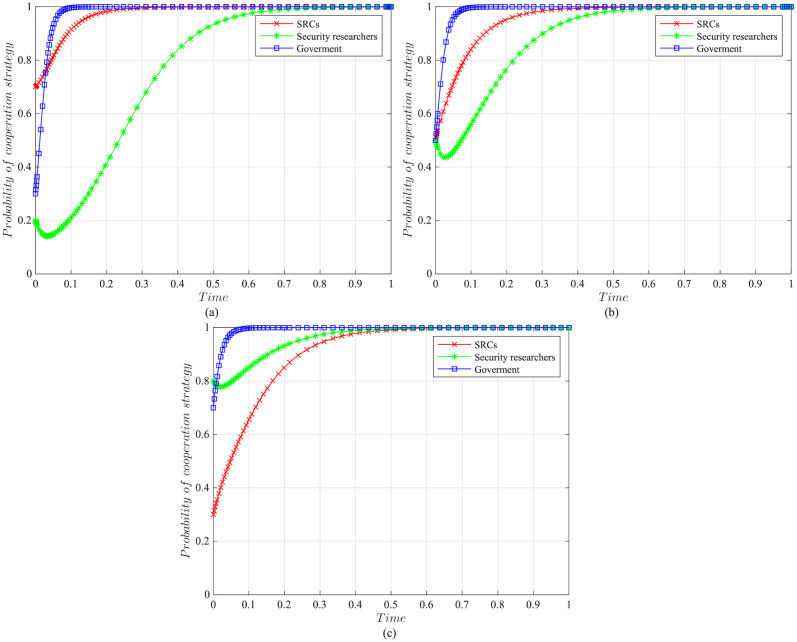
The impact of initial willingness on evolutionary results. (a)Initial willingness is (*x* = 0.7, *y* = 0.2, *z* = 0.3). (b)Initial willingness is (*x* = 0.5, *y* = 0.5, *z* = 0.5). (c)Initial willingness is (*x* = 0.3, *y* = 0.8, *z* = 0.7).

**Fig 7:**
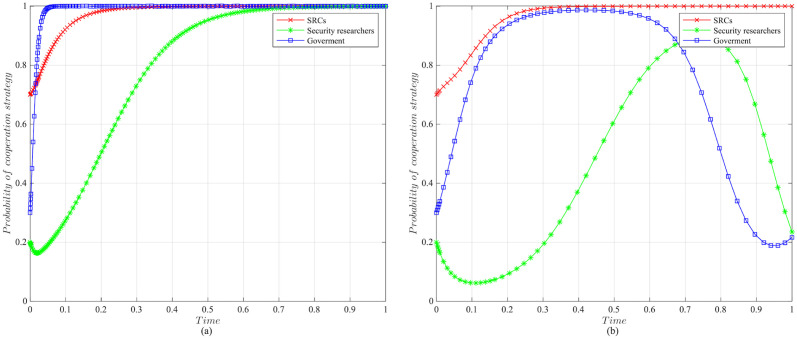
The impact of the government regulatory benefits on evolutionary results. (a)The government regulatory benefits *R* = 150. (b)The government regulatory benefits *R* = 50.

**Fig 8:**
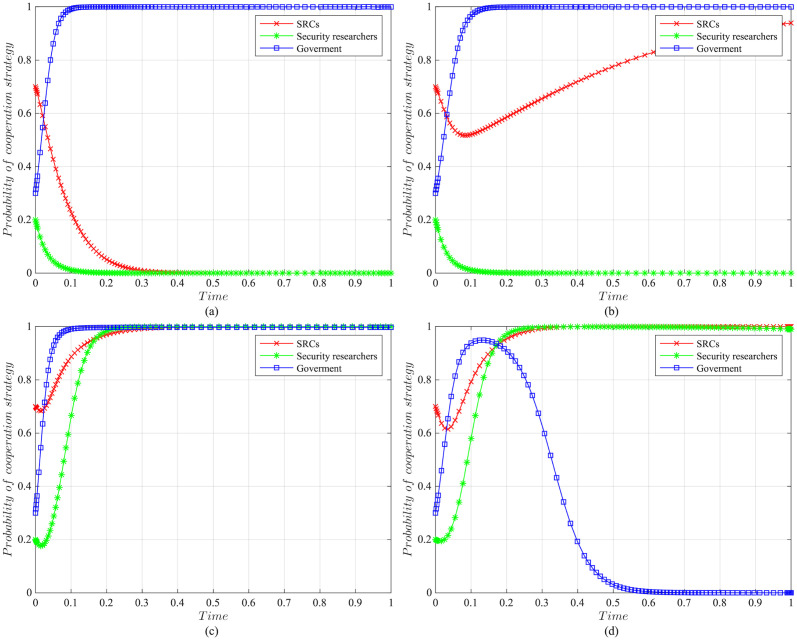
The impact of the government rewards for SRCs on evolutionary results. (a) The government’s rewards for SRCs *A* = 10. (b)The government’s rewards for SRCs *A* = 30. (c)The government’s rewards for SRCs *A* = 50. (d) The government’s rewards for SRCs *A* = 70.

**Fig 9:**
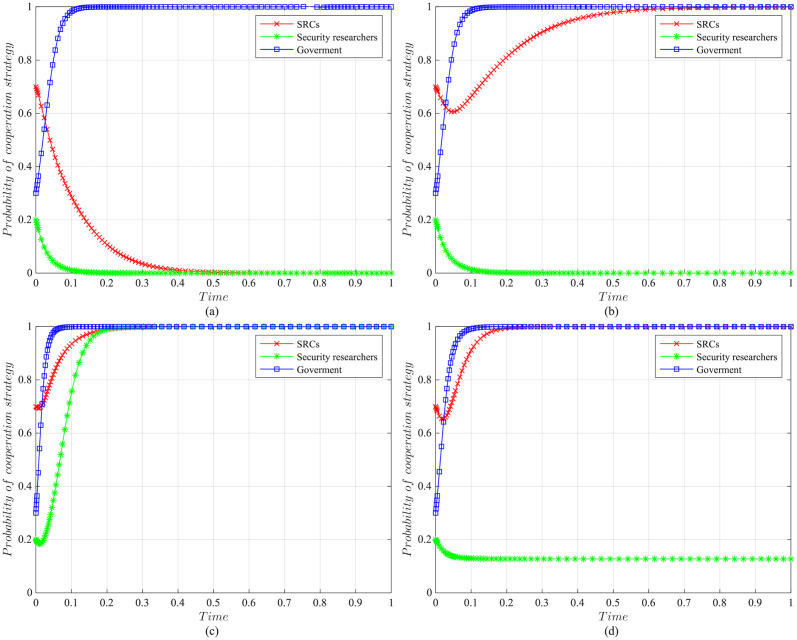
The impact of the government punishments for SRCs on evolutionary results. (a) The government’s punishments for SRCs *K*_1_ = 10. (b)The government’s punishments for SRCs *K*_1_ = 30. (c)The government’s punishments for SRCs *K*_1_ = 50. (d)The government’s punishments for SRCs *K*_1_ = 70.

**Fig 10:**
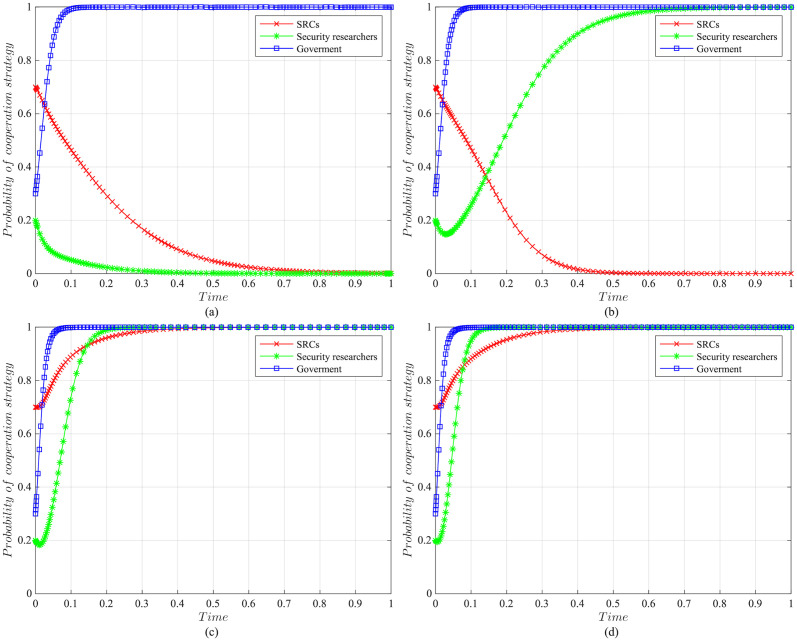
The impact of the government punishments for security researchers on evolutionary results. (a)The government’s punishments for security researchers *K*_2_ = 20. (b)The government’s punishments for security researchers *K*_2_ = 40. (c)The government’s punishments for security researchers *K*_2_ = 60. (d)The government’s punishments for security researchers *K*_2_ = 80.

**Fig 11:**
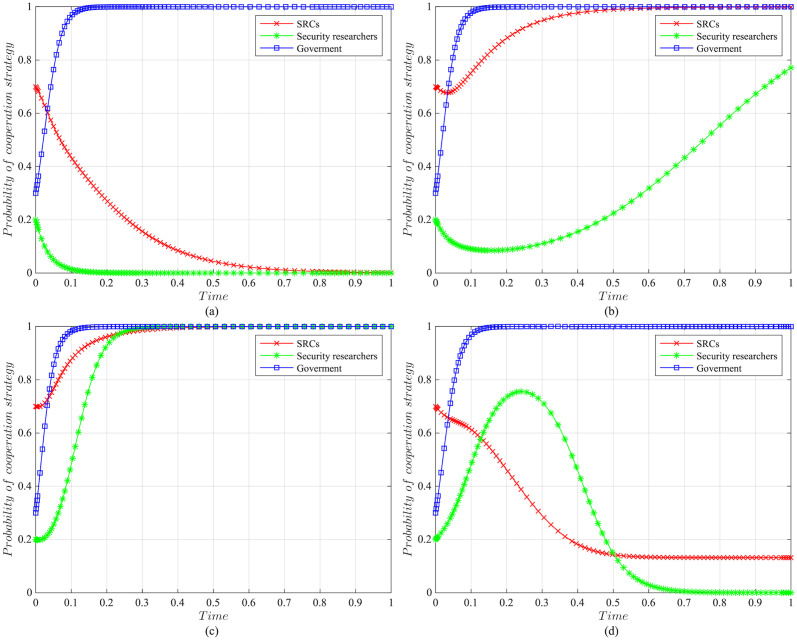
The impact of SRCs’ punishments for security researchers on evolutionary results. (a)SRCs’ punishments for security researchers *F* = 10. (b)SRCs’ punishments for security researchers *F* = 30. (c)SRCs’ punishments for security researchers *F* = 50. (d)SRCs’ punishments for security researchers *F* = 70.

**Fig 12:**
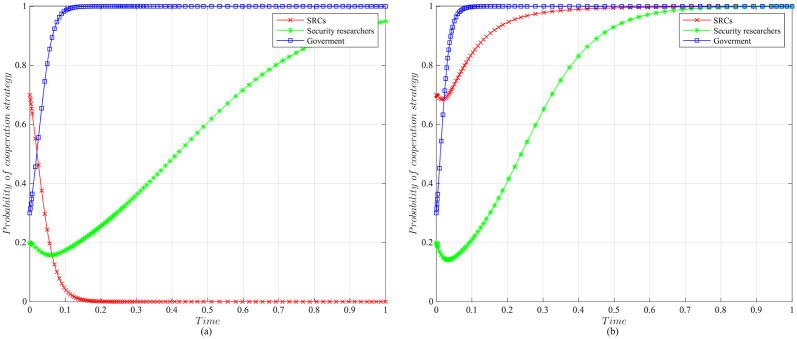
The impact of SRCs’ trust benefits on evolutionary results. (a)The trust benefits *S*_3_ = 5. (b)The trust benefits *S*_3_ = 50.

**Fig 13:**
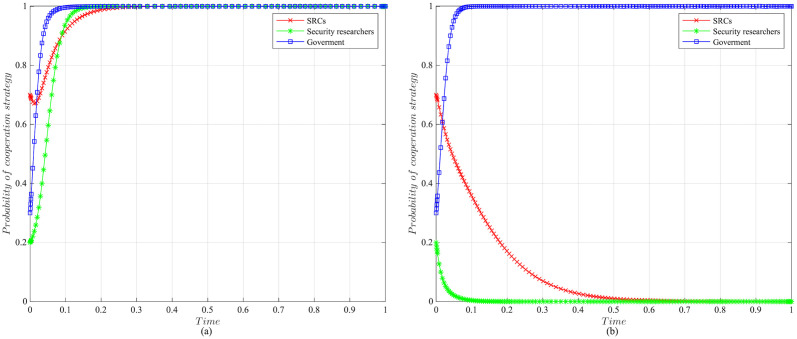
The impact of security researchers’ illegal benefits on evolutionary results. (a)Security researchers’ illegal benefits *P*_2_ = 10. (b)Security researchers’ illegal benefits *P*_2_ = 90.

1. The impact of initial willingness on the system
  Assuming other parameters remain unchanged, setting the initial willingness of the three parties to choose the cooperation strategy is (*x* = 0.7, *y* = 0.2, *z* = 0.3), (*x* = 0.5, *y* = 0.5, *z* = 0.5), (*x* = 0.3, *y* = 0.8, *z* = 0.7), which is the baseline model for the subsequent analysis. The impact of the initial willingness on the evolution system is shown in Fig 6.
  It can be observed that the initial willingness of SRCs, security researchers, and the government has no impact on the system’s evolution strategy, which evolves into the ideal state of collaborative vulnerability disclosure{active management, legal participation, strict regulation}. However, the higher the initial willingness of the three parties to choose cooperative strategies, the faster the system reaches the ideal state of collaborative vulnerability disclosure. Therefore, it can be inferred that in the early stages of SRCs’ establishment, the government and enterprises should actively establish relevant regulatory measures. Specifically, the government should strengthen the regulation of SRCs and security researchers, guiding and regulating their behaviors. Simultaneously, SRCs should enhance the management of security researchers to standardize their behaviors, facilitating the acceleration of reaching the ideal state of collaborative disclosure.
2. The impact of the government regulation benefits on the system
  Based on the initial willingness of (*x* = 0.7, *y* = 0.2, *z* = 0.3), setting *R* to be 150 or 50. The impact of the government regulation benefits on the evolution system is shown in Fig 7.
  When *R* is high, the probability of the three choosing cooperative strategies is higher, and the system stabilizes at the ideal state of collaborative vulnerability disclosure. Moreover, the time of the system to reach the stable state is shortened compared to the baseline model. However, when *R* is relatively low, the government usually weighs the regulatory costs and benefits. When the government realizes that SRCs have initially achieved positive development, it may have lax regulations. In this case, SRCs progressively adopt the negative management strategy, and security researchers who lack the government’s strict regulations choose the illegal participation strategy, leading to an absence of a stable state. Therefore, the government needs to employ various mechanisms to efficiently regulate vulnerability disclosure, reduce regulatory costs and increase regulatory benefits. Additionally, the government should actively establish a positive regulatory image, and improve its reputation, to enhance the willingness of SRCs and security researchers to cooperate with regulation, thereby increasing regulatory benefits.
3. The impact of the government’s rewards and punishments for SRCs on the system
  Based on the above analysis, we investigate the government’s incentive mechanism for SRCs from two aspects, i.e., the reward mechanism and the punishment mechanism. First, assuming other variables remain unchanged, we set the government’s rewards for SRCs *A* to 10, 30, 50, or 70, and the impact on the evolution system is shown in Fig 8.
  When *A* is low, the government’s cost of implementing strict regulatory strategies is low, and the probability of adopting the cooperative strategy is slightly higher compared to the baseline model. For SRCs, the benefits obtained from the government are low, but the costs of adopting the active management strategy are high, leading to a lower probability of the active management strategy. For security researchers, SRCs’ negative management increases their willingness to engage in illegal activities, leading to the system evolving into an ineffective state of {negative management, illegal participation, strict regulation}. When *A* = 50, the system reaches the ideal state of collaborative vulnerability disclosure. When *A* is excessive, although the probability of SRCs adopting the active management strategy increases, the government’s costs increase, reducing the probability of the government’s strict regulation, which leads to the system evolving into an ineffective state of {active management, legal participation, lax regulation}. It is evident that the government’s rewards can incentivize SRCs and security researchers to adopt cooperative strategies, but there exists an effective threshold.
  To further investigate the government’s punishments for SRCs, we set *K*_1_ to 10, 30, 50, or 70, and the results are shown in Fig 9. When *K*_1_ is low, SRCs face to lower punishment costs and higher excess benefits, making them more inclined to adopt the negative management strategy. In this scenario, influenced by SRCs’ negative management, security researchers tend to adopt the illegal participation strategy, ultimately leading to the system evolving into an ineffective state of {negative management, illegal participation, strict regulation}. As *K*_1_ increases, SRCs gradually adopt the active management strategy to reduce punishment costs. When *K*_1_ = 50, punishments for SRCs are sufficient to regulate security researchers’ behavior, and the system evolves into the ideal state of collaborative vulnerability disclosure. However, when *K*_1_ is too high, to avoid excessive punishments, SRCs implement overly strict management of security researchers, which reduces security researchers’ probability of legal disclosure, leading to an ineffective state of {active management, illegal participation, strict regulation}. This suggests that the government can regulate the behaviors of SRCs and security researchers by increasing punishments for SRCs, but should avoid excessive.
4. The impact of the government’s punishments for security researchers on the system
  Based on the above analysis, we investigate the government’s incentive mechanism for security researchers. Assuming other variables remain unchanged, we set the government’s punishments for security researchers *K*_2_ to 20, 40, 60 or 80, and its impact on the evolution of the system is shown in Fig 10.
  When *K*_2_ is low, security researchers’ illegal participation benefits far outweigh the punishments, prompting them to choose the illegal participation strategy. Simultaneously, SRCs adopt negative management due to the lack of legal security researchers, resulting in the system evolving into an ineffective state of {active management, illegal participation, strict regulation}. As *K*_2_ increases, security researchers are initially motivated by the punishment mechanism to engage in legal participation. SRCs are incentivized when *K*_2_ = 60, leading to the system evolving into the ideal state of collaborative vulnerability disclosure. Thereafter, with the increase in *K*_2_, all parties rapidly adopt cooperative strategies, and the time for the system to reach the ideal state is shortened. It is evident that the government’s punishments for security researchers simultaneously incentivize both parties.
5. The impact of SRCs’ punishments for security researchers on the system
  To further investigate the SRCs’ incentive mechanism for security researchers, we set the SRCs’ punishments for security researchers *F* to 10, 30, 50 or 70, and the results are shown in Fig 11.
  When *F* is low, the costs of illegal participation for security researchers are low while the benefits are high, leading them to adopt the illegal participation strategy. Simultaneously, SRCs tend to adopt negative management due to the lack of legal security researchers, resulting in an ineffective state of {negative management, illegal participation, strict regulation}. As *F* increases, security researchers gradually shift towards the legal participation strategy incentivized by stricter punishment mechanisms. And SRCs accelerate the adoption of the active management strategy, leading the system to evolve into the ideal state of collaborative vulnerability disclosure. However, when *F* is excessively high, it may diminish the enthusiasm of security researchers for participation and reduce SRCs’ willingness for active management. Additionally, although the government’s stable strategy remains unaffected, SRCs’ punishment mechanisms provide complementary for government regulation, resulting in a longer time for the government to reach stability compared to the baseline model. It is evident that SRCs implementing reasonable punishment mechanisms can effectively regulate security researchers’ behaviors, enhance their management effectiveness, and to some extent compensate for deficiencies in government regulation.
6. The impact of SRCs’ trust benefits on the system
  To promote cooperation between SRCs and security researchers, we investigate the trust mechanism between SRCs and security researchers. Assuming other variables remain unchanged, we set the trust benefits *S*_3_ to 5 or 50, and its impact on the evolution of the system is shown in Fig 12.
  When *S*_3_ is low, the trust level between SRCs and security researchers is low, and building trust relationships is costly, which leads to SRCs lacking enthusiasm for cooperating with security researchers and tending to choose the negative management strategy. Security researchers may adopt illegal behavior due to the inability to communicate with SRCs in time or to obtain bounties, resulting in the system being unable to evolve into a stable state. When *S*_3_ is high, a high trust level is established between SRCs and security researchers, yielding significant trust benefits, which motivates both parties to adopt the cooperative strategy to maintain long-term collaboration, leading the system to evolve into the ideal stable state of collaborative vulnerability disclosure. This underscores the importance of establishing trust relationships between SRCs and security researchers and its significant implications for the long-term stability of security crowd-testing.
7. The impact of security researchers’ illegal benefits on the system
  Furthermore, to guide security researchers to engage in legal participation, we investigate the publicity and training mechanisms for them. Assuming other variables remain unchanged, we set the security researchers’ illegal benefits *P*_2_ to 10 or 90, and its impact on the evolution of the system is shown in Fig 13.
  When *P*_2_ is low, security researchers actively participate in security crowd-testing to earn bounties, increasing the probability of legal participation. Simultaneously, SRCs manage actively, ultimately leading the system to evolve into a stable state of collaborative vulnerability disclosure. However, with the increase in *P*_2_, higher illegal benefits induce security researchers to adopt the illegal participation strategy rather than the legal one. In this case, the security crowd-testing market environment deteriorates, making it challenging for SRCs to achieve expected benefits, reducing their enthusiasm for active management, which causes the system to evolve into an ineffective state of {negative management, illegal participation, strict regulation}. This indicates that excessive illegal benefits significantly hinder the enthusiasm for vulnerability disclosure by SRCs and security researchers, and it is necessary to take publicity and training mechanisms to guide their behavior.

## 6 Discussion

### 6.1 Conclusions

Security crowd-testing has reduced the barriers to the vulnerability disclosure process but has also brought about issues like non-compliant vulnerability disclosure and conflicts of interest. Therefore, to promote collaborative disclosure among various stakeholders, investigating the regulatory mechanisms of vulnerability disclosure behaviors in security crowd-testing is essential. In view of this, based on evolutionary game theory, this paper explores the evolutionary process of strategies for SRCs, security researchers, and the government, as well as the corresponding stable states. Subsequently, numerical simulations are conducted by MATLAB to investigate the impact of key parameters on evolutionary stability and propose targeted regulatory mechanisms.

Differing from previous studies, we examine the evolution of vulnerability disclosure behaviors in multi-agent interactions from a management perspective and identify the following conclusions. Firstly, in terms of the factors and mechanisms impacting the evolutionary game system’s steady state, the initial willingness of SRCs, security researchers, and the government has no impact on the system’s stable evolutionary strategy, which evolves to the stable state of collaborative vulnerability disclosure {active management, legal participation, strict regulation}. The higher their initial willingness to adopt cooperative strategies, the faster the system reaches this equilibrium, which was consistent with the conclusion of Zhou et al. [51]. However, when government regulatory benefits are low, the system cannot reach a stable state. Secondly, in terms of the government’s incentive mechanisms for SRCs, increasing rewards and punishments can incentivize SRCs and security researchers to adopt cooperative strategies. However, excessive punishments result in high regulatory costs that are unfavorable for the government to implement strict regulation, and excessive rewards induce SRCs to adopt overly stringent management measures, thereby diminishing the motivation of security researchers, both of which hinder collaborative disclosure. Chen et al. [50] constructed a similar evolutionary game model for government subsidies, which also concluded that excessive government subsidies are detrimental to reaching the system’s steady state. Thirdly, in terms of the government’s incentive mechanisms for security researchers, different from the findings of Zhao et al. [56], we find that compared to incentive mechanisms for SRCs, the government’s increasing punishments for security researchers are more effective, which can encourage SRCs to adopt cooperative strategies by regulating security researchers’ behaviors. Fourthly, in terms of SRCs’ incentive mechanisms for security researchers, while SRCs’ increasing punishments for security researchers may decelerate the government reaching a stable state, it can compensate for the government’s deficiencies in sole regulation by forming a society-wide co-regulatory system as mentioned by Chen et al. [53], but excessive punishments should be avoided. Fifthly, in terms of the trust mechanism between SRCs and security researchers, increasing trust benefits or reducing illegal benefits can enhance the willingness of SRCs and security researchers to choose cooperative strategies, which plays an important role in promoting the system to reach the ideal state of collaborative disclosure. It is consistent with Chen et al. [57] who used evolutionary game theory to argue that building trust relationships is a crucial part of public crisis governance.

**Fig 14:**
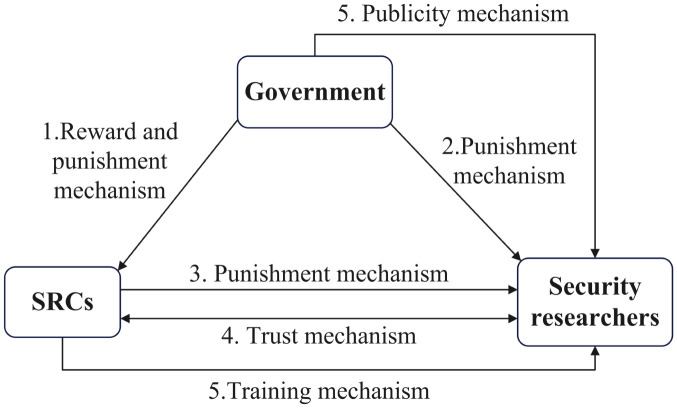
Proposed regulatory mechanisms.

Combining previous research and based on the analysis results of this paper, we optimize existing regulatory mechanisms and propose new regulatory mechanisms from the perspective of the new model of security crowd-testing. In response to different regulators and those being regulated, incentive mechanisms including the reward mechanism, the punishment mechanism, the trust mechanism, and the publicity and training mechanism have been proposed, as shown in Fig 14.

1. The government’s reward and punishment mechanism for SRCs
  The government should set appropriate reward levels based on the situation of SRCs and security researchers, and dynamically adjust the reward mechanism to achieve the dual goals of reducing regulatory costs and maximizing the effectiveness of the reward mechanism. Meanwhile, to swiftly achieve the goal of collaborative vulnerability disclosure, the government should adopt the regulatory mechanism as “punishments primarily, rewards complementary”, while setting scientific punishment levels to achieve effective regulation without diminishing the enthusiasm of participants in vulnerability disclosure.
2. The government’s punishment mechanism for security researchers
  The government should clarify the policy provisions and industry norms of security crowd-testing and implement strict punishment mechanisms for security researchers. To intensify punishments for security researchers engaged in illegal activities, the government should implement measures including administrative sanctions, reputational punishments, and notification of illegal activities, to fundamentally reduce or prevent the illegal participation of security researchers, optimizing the environment of the security crowd-testing market.
3. SRCs’ punishment mechanism for security researchers
  To incentivize security researchers to participate legally, SRCs must enhance the platform’s relevant regulations in accordance with the government’s policies, including clearly defining rights and responsibilities, establishing responsibility boundaries, and clarifying illegal activities. Moreover, SRCs should promptly detect and punish illegal security researchers based on platform rules, including fines, revoking credits, and account suspensions. This supplements government regulation, establishing a collaborative regulatory system between the government and SRCs.
4. The trust mechanism between SRCs and security researchers
  SRCs should proactively implement diverse trust mechanism through multiple channels, including establishing communication platforms, promoting multi-channel communication, and implementing vulnerability disclosure credit systems, to actively build a trust framework enhancing the long-term effectiveness of security crowd-testing vulnerability disclosure.
5. The publicity and training mechanism for security researchers
  The government should establish a comprehensive publicity mechanism, including policy advocacy, the establishment of publicity platforms, and the organization of regular publicity events, to clarify the severe harm and consequences of illegal activities, reducing security researchers’ illegal participation willingness. Additionally, SRCs should establish a training mechanism, including routine training, online training, and periodic evaluations, to enhance security researchers’ capabilities and reduce their illegal benefits, which can optimize the security crowd-testing environment to promote collaborative vulnerability disclosure.

### 6.2 Limitations and future work

This paper provides a theoretical foundation and practical recommendations for regulatory mechanisms of participants’ vulnerability disclosure behaviors in security crowd-testing. There are still worthy viewpoints to further study. On one hand, vulnerability disclosure in security crowd-testing is a complex process involving multiple stakeholders, while we only focus on the core participants involved in vulnerability disclosure and do not consider various types and characteristics of participants, such as social public and the media, etc., or considering the homogeneity or heterogeneity of SRCs or security researchers as well. On the other hand, the security crowd-testing environment is not sufficiently detailed, which considers the cooperation between SRCs and security researchers, as well as between enterprises and SRCs. In fact, there is intense competition among enterprises, SRCs and security researchers. In future research, it may be worthwhile to analyze vulnerability disclosure issues from a competitive perspective, considering the preferences and attributes of different participants.

## Supporting information

S1 File (ZIP)
